# Mast Cells Interact with Endothelial Cells to Accelerate In Vitro Angiogenesis

**DOI:** 10.3390/ijms18122674

**Published:** 2017-12-13

**Authors:** Devandir Antonio de Souza Junior, Vivian Marino Mazucato, Ana Carolina Santana, Constance Oliver, Maria Celia Jamur

**Affiliations:** Department of Cell and Molecular Biology and Pathogenic Bioagents, Ribeirão Preto Medical School, University of São Paulo, 14.049-900 Ribeirão Preto, SP, Brazil; dasjunior@hotmail.com (D.A.d.S.J.); vivianmazucato@hotmail.com (V.M.M.); carousantana@gmail.com (A.C.S.); coliver@fmrp.usp.br (C.O.)

**Keywords:** mast cells, angiogenesis, endothelial cells, tube formation assay, co-culture, gap junction

## Abstract

Angiogenesis is a complex process that involves interactions between endothelial cells and various other cell types as well as the tissue microenvironment. Several previous studies have demonstrated that mast cells accumulate at angiogenic sites. In spite of the evidence suggesting a relationship between mast cells and angiogenesis, the association of mast cells and endothelial cells remains poorly understood. The present study aims to investigate the relationship between mast cells and endothelial cells during in vitro angiogenesis. When endothelial cells were co-cultured with mast cells, angiogenesis was stimulated. Furthermore, there was direct intercellular communication via gap junctions between the two cell types. In addition, the presence of mast cells stimulated endothelial cells to release angiogenic factors. Moreover, conditioned medium from the co-cultures also stimulated in vitro angiogenesis. The results from this investigation demonstrate that mast cells have both direct and indirect proangiogenic effects and provide new insights into the role of mast cells in angiogenesis.

## 1. Introduction

Angiogenesis depends on endothelial cell proliferation and migration. Blood vessel formation is also dependent on the interaction of endothelial cells with other cell types, as well as with the tissue microenvironment [1,2,3]. Therefore, it is essential to understand the interaction of endothelial cells with other cell types and how this interaction can activate endothelial cells during blood vessel formation. Throughout angiogenesis, these various cell types communicate via a complex network of intercellular signaling pathways, mediated by cytokines, receptors and surface adhesion molecules [2,4,5,6].

Cell-to-cell communication is critical in the control and coordination of vascular function [7,8,9]. The gap junction is the main mediator of cell-to-cell communication. It is formed by connexins [10,11,12], a family of transmembrane proteins that interact to form connexons that act as channels which directly connect the cytoplasm of adjacent cells, allowing the passage of current and small signaling molecules [8,11]. Several studies have suggested that gap junctions and connexins may play an important role during physiological or pathological processes associated with angiogenesis [9,13,14]. Furthermore, gap junctions have been identified at areas of contact between immune cells and endothelial cells. Gap junctions have also been implicated in immune cell trans-endothelial migration and in the contact between eosinophils and tissue resident cells during inflammatory reactions [15,16,17].

Recently, the interactions between immune cells such as macrophages, lymphocytes, neutrophils, and endothelial cells during angiogenesis have been described [1,18,19]. However, the interaction between mast cells and endothelial cells during angiogenesis has not been completely delineated. Mast cells are immune cells that are derived from myeloid lineage cells in the bone marrow [20,21,22]. They are found associated with connective tissue throughout the body, particularly in locations that are in close contact with the external environment [23,24,25]. Mast cells have been shown to be associated with blood vessels and they accumulate near sites of new capillary formation, such as in atherosclerosis, hemangioma development, and tumorigenesis [26,27,28,29,30,31,32,33]. Notably, mast cell accumulation is correlated with increased neovascularization, tumor aggressiveness, and metastatic spread [34,35,36,37]. Previous studies have focused on the role of mast cells in enhancing angiogenesis through the release of pro-angiogenic factors [35,38,39,40]. However, the involvement of mast cells in angiogenesis is complex and not completely understood. The present study was undertaken to investigate the interaction of mast cells and endothelial cells in angiogenesis. During in vitro angiogenesis [41,42], when mast cells were co-cultured with endothelial cells, an association between mast cells and endothelial cells was observed. Furthermore, this association induced the release of pro-angiogenic factors which accelerated angiogenesis.

## 2. Results

### 2.1. Mast Cells Accelerate In Vitro Angiogenesis

**Figure 1:**
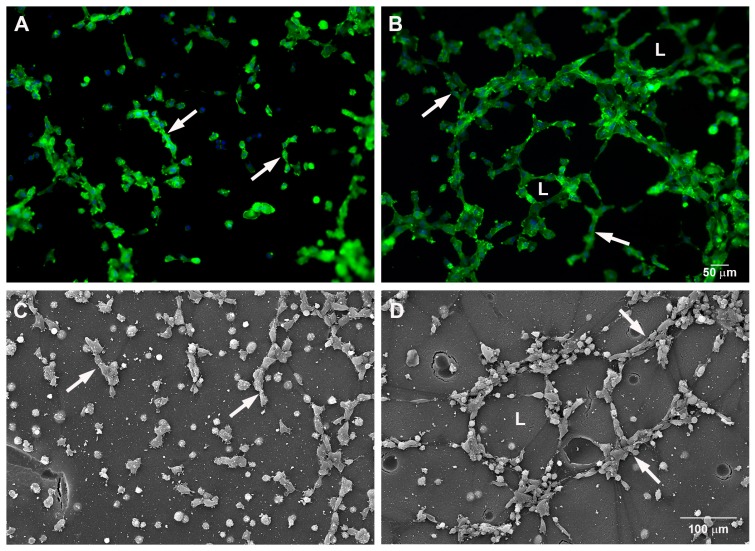

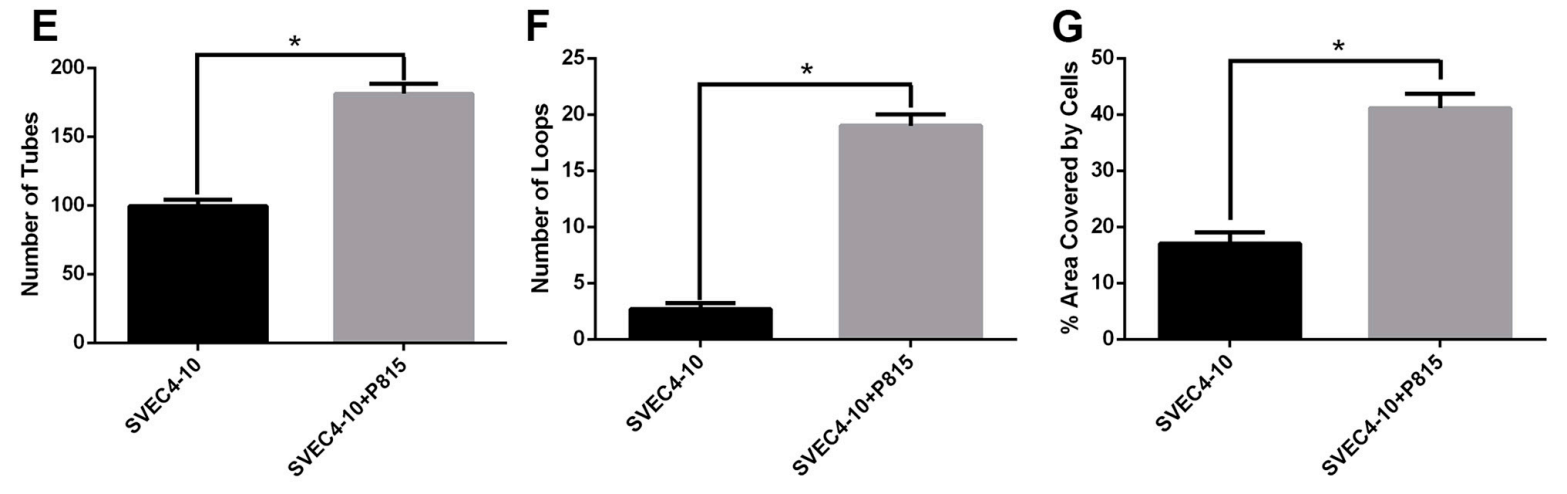
Mast cells induce tube and loop formation. (**A**,**C**) SVEC4-10 cells; (**B**,**D**) SVEC4-10 cells co-cultured with P815 mast cells. Tubes (arrows) and loops (L) were increased in the co-cultures after 5 h. (**A**,**B**) Cells were stained with phalloidin conjugated to Alexa 488; the nuclei were counterstained with DAPI (4′, 6-Diamine-2′-phenylindole dihydrochloride); (**C**,**D**) scanning electron microscopy; (**E**–**G**) The graphs show the average number of tubes (**E**) and loops (**F**) and the area covered by cells (**G**) after 5 h of culture. The structures were quantified using Wimasis WimTube. Data are presented as mean ± SD from five independent experiments. * *p* ≤ 0.05.

To evaluate the ability of mast cells to induce angiogenesis, the mouse endothelial cell line SVEC4-10 and the murine mast cell line P815 were co-cultured using an in vitro angiogenesis assay system. Tube formation was evaluated after five hours of co-culture by actin staining as well as by scanning electron microscopy. Tube formation and sprouting by endothelial cells was increased in the presence of mast cells (Figure 1A–D). A quantitative analysis of the co-culture showed that the percentage of tubes and loops was higher, 85 ± 12% and 842 ± 108% respectively, when SVEC4-10 cells were cultured together with the mast cells (Figure 1E,F). Also, the area covered by cells was significantly higher in the co-cultures, 233 ± 33%, (Figure 1G), indicating an increase in cell spreading.

**Figure 2:**
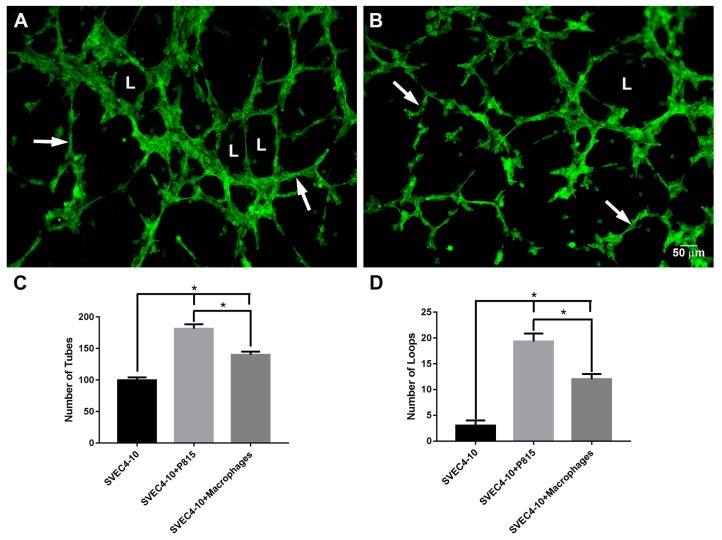
Mast cells are more effective in inducing tube formation than macrophages. (**A**) SVEC4-10 cells co-cultured with P815 mast cells; (**B**) SVEC4-10 cells co-cultured with macrophages. A significant increase in the number of tubes (arrows) and loops (L) was observed during co-culture of endothelial cells with mast cells when compared with co-cultures of endothelial cells with macrophages. (**A**,**B**) Cells were stained with phalloidin conjugated to Alexa 488; (**C**,**D**) The graphs show the average number of tubes and loops after 5 h of culture. The structures were quantified using Wimasis WimTube. Data are presented as mean ± SD from five independent experiments. * *p* ≤ 0.05.

Since it is known that macrophages can also enhance angiogenesis [43,44,45], endothelial cells were co-cultured with macrophages in order to verify the results seen with mast cells. Co-culture of endothelial cells with mast cells (Figure 2A) was more effective in inducing tube and loop formation than co-culture of SVEC4-10 cells with macrophages (Figure 2B). The percentage of tubes increased 43 ± 9% (Figure 2C) and the percentage of loops increased 462 ± 88% (Figure 2D) in the co-cultures of endothelial cells with mast cells when compared with the co-cultures of endothelial cells with macrophages. The results of the tube formation assays showed that mast cells are more effective in promoting in vitro angiogenesis than macrophages.

### 2.2. Mast Cells and Endothelial Cells Are Associated

**Figure 3:**
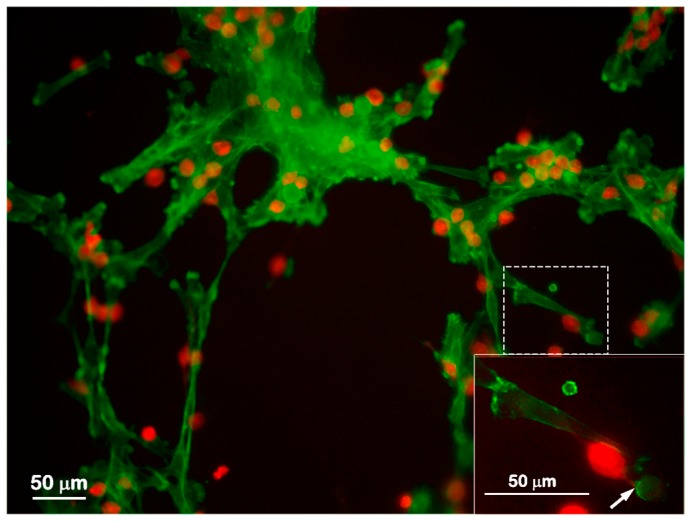
Mast cells are in close approximation to endothelial cells. SVEC4-10 cells co-cultured with P815 mast cells. SVEC4-10 cells were labeled with CellTracker™ Green CMFDA and P815 mast cells were labeled with CellTracker™ Red CMTPX. After 5 h of co-culture, SVEC4-10 cells (green) and P815 mast cells (red) are associated. Mast cells are in contact with endothelial cells (arrow). Inset: High magnification of the area delimited by dotted line. Image is representative of four independent experiments.

Since the presence of mast cells accelerated tube and loop formation by endothelial cells, the association between these two cell types was further investigated. SVEC4-10 cells were labeled with CellTracker™ Green CMFDA (5-chloromethylfluorescein diacetate) and P815 mast cells were labeled CellTracker™ Red CMTPX (4(or 5)-(4-(chloromethyl)benzamido)-2-(1,2,2,4,8,10,10,11-octamethyl-1,2,10,11-tetrahydropyrano[3,2-g:5,6-g′]diquinolin-13-ium-6-yl)benzoate). After 5 h of co-culture, P815 mast cells were in close association with SVEC4-10 cells (Figure 3) especially in the loops. The presence of mast cells in close association with endothelial cells during the co-cultivation suggests that mast cells play a critical role in vitro angiogenesis.

**Figure 4:**
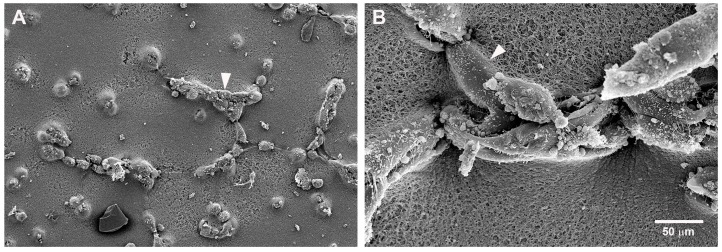

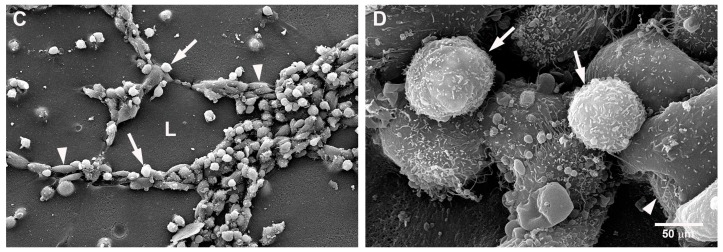
Mast cells and endothelial cells associate during the tube formation assay. (**A**,**B**) SVEC4-10 cells; (**C,D**) SVEC4-10 cells co-cultured with P815 mast cells. (**A**,**B**) In cell cultures without mast cells, the endothelial cells form only incomplete loops (arrowheads); (**C**,**D**) During co-culture SVEC4-10 endothelial cells (arrowheads) and mast cells (arrows) are associated especially in the loops (L). The endothelial cells are spread on the substrate (arrowheads) while P815 mast cells remain rounded (arrows). Cells were cultured for 5 h prior to assay. Scanning electron microscopy images are representative of four independent experiments.

This association between mast cells and endothelial cells was confirmed by scanning electron microscopy (Figure 4). In the co-culture, most of the mast cells were in contact with endothelial cells. They could also be seen in the loops, which are the final step in tube formation (Figure 4C,D). The morphological response of the two cell types to culture on Geltrex^®^ was different. SVEC4-10 cells spread on the Geltrex^®^ while the P815 mast cells remained rounded (Figure 4C,D). Mast cells also remained rounded when cultured on Geltrex^®^ even in the absence of endothelial cells. 

### 2.3. Gap Junctions Connect Endothelial Cells and Mast Cells

**Figure 5:**
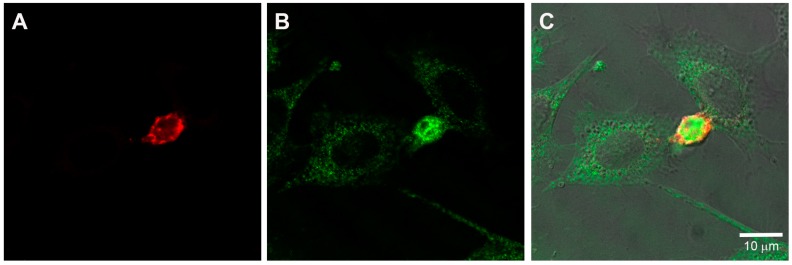
Connexin 43 is expressed in P815 mast cells and endothelial cells. (**A**) P815 mast cells were labeled with CellTracker™ Red CMTPX; (**B**) After 5 h of co-culture with SVEC4-10 cells, the cultures were immunostained with antibody against connexin 43 (green); (**C**) Merge of A and B. Images are representative of three independent experiments.

The presence of gap junctions between endothelial cells and mast cells was investigated by immunostaining the co-cultures for connexin 43. The results showed that both mast cells and endothelial cells expressed this protein and that connexin 43 was present throughout the cells including cellular projections, as well as the area of contact between mast cells and endothelial cells (Figure 5).

**Figure 6:**
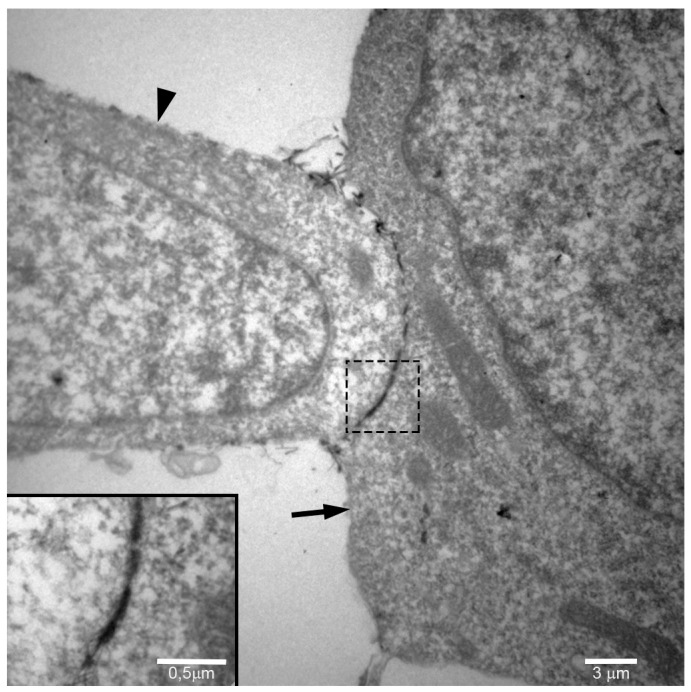
Gap junctions are present between mast cells and endothelial cells during in vitro angiogenesis. Transmission electron microscopy demonstrates that the space between the mast cell (arrow) and the endothelial cell (arrowhead) is filled with lanthanum, indicative of a gap junction. Inset: High magnification of the area delimited by dotted line.

The association between mast cells and endothelial cells was further investigated by transmission electron microscopy. Mast cells and endothelial cells were co-cultured on Geltrex^®^ for 5 h and then processed for transmission electron microscopy. An intercellular space (7–15 Å) between the endothelial cells and mast cells filled with lanthanum, which is indicative of gap junctions, was evident (Figure 6). As suggested by the immunostaining, the results of the transmission electron microscopy indicate a direct intercellular communication, via gap junctions, between mast cells and endothelial cells during angiogenesis.

### 2.4. Angiogenic Factors Are Released by Endothelial Cells in the Presence of Mast Cells

Since co-culture of mast cells with endothelial cells induced tube formation, it was of interest to investigate which angiogenic factors were released during the co-culture. The supernatants from the co-cultures of SVEC4-10 cells with P815 mast cells or from cultures of SVEC4-10 cells alone were analyzed using a proteome profiler array. In the presence of mast cells, SVEC4-10 cells released 33 more angiogenic factors than SVEC4-10 cells alone (Table 1; Figure S1). Analysis of the supernatants from the co-culture showed that the mast cells did not degranulate. There was no release of β-hexosaminidase activity nor were mMCPs (mouse mast cell proteases) 5 and 6 (Figure S2) detected in the supernantants. These results provide evidence that the presence of mast cells stimulates the release of angiogenic factors from the endothelial cells.

### 2.5. Conditioned Medium from the Co-Cultures Increases Tube Formation

**Figure 7:**
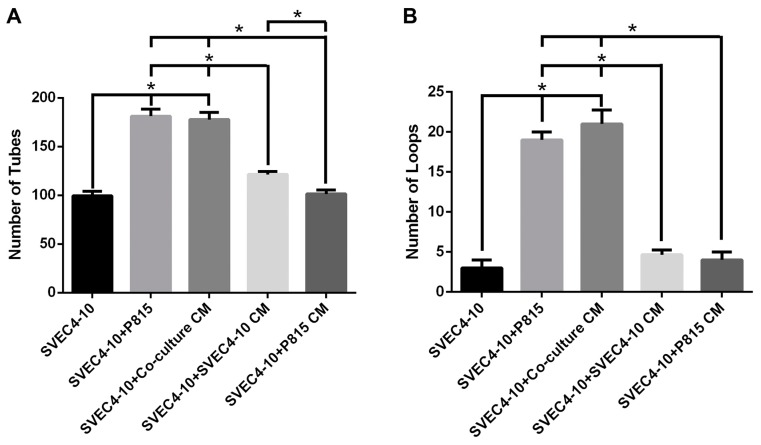
Conditioned medium from the co-culture increases in vitro angiogenesis. The conditioned medium from the co-culture of SVEC4-10 cells and P815 mast cells demonstrated the same ability as co-culture with mast cells to increase in vitro angiogenesis. (**A**,**B**) The conditioned media from SVEC4-10 induced a slight increase in tubes (**A**) but not in loops. The graphs show the average number of tubes and loops after 5 h of culture. The structures were quantified using Wimasis WimTube. Data are presented as mean ± SD from five independent experiments. CM (Conditioned media) * *p* ≤ 0.05.

Since co-culture of endothelial cells with mast cells increased tube and loop formation, it was of interest to determine if the physical presence of mast cells was necessary to stimulate in vitro angiogenesis. Therefore, endothelial cells were cultured with conditioned media from various culture conditions (Figure 7). When SVEC4-10 cells were cultured on Geltrex^®^ in the presence of conditioned medium from SVEC4-10 cells, there was a significant increase in tube, but not loop formation. However, there was an even more significant increase in both tube and loop formation when SVEC4-10 cells were cultured on Geltrex^®^ in the presence of conditioned medium from the co-cultures of endothelial cells and mast cells. Furthermore, when SVEC4-10 cells were cultured on Geltrex^®^ in the presence of conditioned medium from P815 mast cells, there was no increase in tube or loop formation. These results indicate that mast cells themselves and/or the angiogenic mediators released by endothelial cells in the presence of mast cells both stimulate angiogenesis.

## 3. Discussion

The present study provides evidence of a direct interaction between endothelial cells and mast cells that results in the release of angiogenic factors from endothelial cells which then accelerate in vitro angiogenesis. Furthermore, conditioned medium from the co-culture of endothelial cells and mast cells contains the factors necessary to stimulate angiogenesis in vitro. These results demonstrate a critical role for mast cells in angiogenesis.

As shown in the present study, during in vitro angiogenesis, the mast cells were in close proximity to the endothelial cells where they were largely associated with loops. This is consistent with the localization of mast cells in vivo, where mast cells are normally found adjacent to blood vessels [46,47]. Angiogenic factors induce endothelial cells to sprout and form branches that split in two, increasing the area of the new vessels. This branching also facilitates fusion of the endothelial cells in a process called anastomosis [44,48,49,50]. In the developing hindbrains of zebrafish, macrophages appear to form bridges between endothelial cells aiding in cell fusion and promoting anastomosis between endothelial cells [51]. The present study provides evidence that mast cells could also act as bridges giving physical support and establishing contact between endothelial cells during vascular anastomosis.

The current investigation also demonstrates that mast cells and endothelial cells can form gap junctions. Ultrastructural analysis indicated a direct intercellular communication, via gap junctions, between mast cells and endothelial cells during angiogenesis. Coordination and control of vascular function is dependent on direct intercellular communication via gap junctions [52,53]. Gap junctions directly connect the cytoplasm of adjacent cells allowing for the passage of small signaling molecules and current. A gap junction is composed of two connexons integrated into the plasma membranes of the connected cells forming a channel [54,55]. Each connexon is composed of six connexins. It is the connexins which modulate specific cell-to-cell signaling pathways. The various connexins differ in their molecular selectivity as well as in their subcellular localization. In addition, Cx43 (connexin-43) seems to be essential for coordination of endothelial cell proliferation and migration in the vasculature [8,52,56,57]. Vliagoftis et al. demonstrated that mast cells express Cx43 and Cx32, and that Cx43 is associated with the plasma membrane [58]. Recent studies have shown that mast cells can also interact directly with fibroblasts, forming gap junctions between mast cells and fibroblasts. This allows direct communication between the two cell types, stimulating fibroblast proliferation, myofibroblast differentiation, and collagen lattice contraction [59,60,61,62]. The physical association of mast cells and endothelial cells as well as the direct intercellular communication via gap junctions observed in this study, provides another mechanism in which mast cells may affect angiogenesis.

Mast cells produce and release a large number of mediators with diverse biological activities. Previous studies have demonstrated that mast cell proteases are among the main factors responsible for stimulating angiogenesis [29,63,64]. However, when the activity of β-hexosaminidase and the expression and activity of tryptase and chymase were analyzed in supernatants from co-cultures of mast cells and endothelial cells, no β-hexosaminidase activity or tryptase or chymase expression or activity was detected in the supernatants from these experiments. Additionally, the cells were co-cultured for only five hours. This time is not sufficient for the synthesis and release of newly synthesized mediators, such as growth factors and cytokines [22,23]. These data demonstrate that in co-culture with endothelial cells, the mast cells did not release mast cell proteases or other preformed mediators.

In addition, the current investigation showed that the presence of mast cells stimulated the endothelial cells to release a wide range of angiogenic factors. The levels of the pro-angiogenic cytokines angiogenin, endostatin, IGFBP-3 (Insulin-like growth factor binding protein-3), and MCP-1 (Monocyte chemoattractant protein-1) detected in cultures of SVEC4-10 cells alone, increased when the SVEC4-10 cells were co-cultured with mast cells.

Endothelial cell spreading was accelerated in the co-cultures of mast cells and endothelial cells as well as when the endothelial cells were cultured in conditioned medium from the co-cultures. This may be due to the presence of various other proteins/cytokines that were detected in the supernatant from the co-cultures that were not present in the culture media of endothelial cells alone. Some of these proteins/cytokines are directly related to cell proliferation and migration such as SDF-1 (Stromal cell-derived factor 1), and trombopondin-2 [65]. Therefore, the release of various cytokines/proteins related to adhesion and migration when endothelial cells are in the presence of mast cells may explain the increased spreading of endothelial cells, as well as the stimulation of tube and loop formation during co-cultivation of endothelial cells with mast cells or with conditioned medium from the co-cultures.

## 4. Materials and Methods

### 4.1. Cells

The murine endothelial cell line SVEC4-10 (CRL-2181) was purchased from the American Type Culture Collection (ATCC; Manassas, VA, USA). The P815 mast cells (mouse lymphoblast-like mastocytoma cell line) [66,67,68,69] was purchased from the Rio de Janeiro Cell Bank (Rio de Janeiro, Brazil). Both cell lines were maintained in Dulbecco’s Modified Eagle’s Medium (DMEM) plus 10% heat inactivated fetal bovine serum (FBS). The murine lung macrophage cell line AMJ2-C11 (ATCC^®^ CRL-2456™) was cultivated in DMEM plus 5% heat inactivated fetal bovine serum. All cell lines were cultured at 37 °C in a humidified environment containing 5% CO_2_ in air. All reagents used for cell culture were purchased from Thermo Fisher Scientific (Invitrogen/Thermo Fisher Scientific, Waltham, MA, USA).

### 4.2. In Vitro Angiogenesis: Tube Formation Assay

The tube formation assay measures the ability of endothelial cells to form tubes which are capillary-like structures. In order to perform this assay, 10 μL of Geltrex^®^ (Invitrogen, Thermo Fisher Scientific) was added to the wells of μ-slides Angiogenesis^®^ (IBIDI, Martinsried, Germany) and allowed to solidify at 37 °C for 30 min. After the gel solidified, SVEC4-10 cells (1 × 10^4^) were added in 50 μL of DMEM supplemented with 10% FBS. For co-culture experiments, the cells were cultured at a ratio of 1 P815 mast cell or AMJ2-C11 macrophage to 2 SVEC4-10 cells. In the experiment using conditioned media, the media were collected after tube formation assay as described above and used in a new tube formation assay. The following conditioned media were used in tube formation assays: Conditioned medium from the co-culture of mast cells and endothelial cells; Conditioned medium from the culture of SVEC4-10 cells alone; Conditioned medium from the culture of P815 mast cells alone.

The cells were incubated at 37 °C in a humidified atmosphere containing 5% CO_2_ in air for 5 h and then analyzed. The following parameters were used for quantification [70,71,72].

Tubes were considered to be a tubular structure that goes from one branching point to another branching point or to a loose end. Loops are enclosed (or almost enclosed) areas inside the tubes that fulfill roundness conditions.

The images were acquired using a Nikon Eclipse TE2000-U microscope (Nikon USA, Melville, NY, USA) equipped with a 10× objective. The measurements are expressed in pixels (800 × 600) where 1 pixel equals 0.069 mm^2^. WimTube (Wimasis Image Analysis, Munich, Germany; www.wimasis.com/en/products/13/WimTube) was used to analyze and quantify tube formation.

### 4.3. CellTracker™ Cell Labelling

SVEC4-10 cells were labeled with CellTracker™ Green CMFDA and P815 mast cells were labeled with CellTracker™ Red CMTPX (Molecular Probes, Thermo Fisher Scientific). The cells were incubated for 30 min at 37 °C in DMEM without serum, but containing 25 μg/mL of either CellTracker™ green or red. After labeling, the cells were incubated for an additional 30 min at 37 °C in DMEM with 10% heat inactivated fetal bovine serum. The cells were used in the tube formation assay as described above.

### 4.4. Immunofluorescence

To determine the presence of connexin 43 in endothelial cells and mast cells, the cells were fixed with 2% formaldehyde (Electron Microscopy Sciences, Hatfield, PA, USA), permeabilized with 0.1% Triton X-100 in PBS at room temperature, blocked with PBS containing 1% BSA, and incubated with rabbit anti-mouse connexin 43 (1:50) (ATB T1046, Ameritech Biomedicines, Pflugerville, TX, USA) for 1 h. Following incubation, cells were washed, and incubated with goat anti-rabbit IgG conjugated with Alexa 488 (1:1000) (Molecular Probes, Thermo Fisher Scientific), washed and analyzed using a LEICA TCS-NT SP5 laser scanning confocal microscope (Leica Microsystems; Heidelberg, Germany).

### 4.5. Fluorescent Labeling

Cells were fixed with 2% formaldehyde (Electron Microscopy Sciences), permeabilized with 0.3% Triton X-100 in PBS for 10 min, washed, and then incubated with 2.6 U/mL Phalloidin-Alexa 488 (Molecular Probes, Thermo Fisher Scientific) for 30 min. Nuclei were counterstained with 4,6-diamidino-2-phenylindole (DAPI; 1:500). The samples were washed in PBS and then observed with an inverted fluorescence microscope (Nikon Eclipse TE2000-U, Nikon USA) and the images acquired using a Nikon DS-1QM digital camera (Nikon USA).

### 4.6. Expression Profile of Angiogenesis Related Proteins

The expression profile of angiogenesis related proteins was analyzed using the Proteome Profiler™ Mouse Angiogenesis Antibody Array (R & D Systems Inc., Minneapolis, MN, USA). After the tube formation assay, the supernatants from SVEC4-10 cell cultures or co-cultures of SVEC4-10 cells and P815 mast cells were collected and processed following the manufacturer’s instructions. Briefly, the membrane containing immobilized antibodies to angiogenesis related proteins was incubated with the supernatants. Bound protein was detected with a biotinylated antibody cocktail followed by streptavidin conjugated to horseradish peroxidase. Membranes were washed and developed using the ECL™ Western Blotting Detection Reagent, RPN2106 (GE Healthcare, Piscataway, NY, USA). The data is expressed as the mean of two assays.

### 4.7. Electron Microscopy

For electron microscopy assays, Geltrex^®^ (100 μL) was placed on 13-mm-round coverslips coated with Biobond (Electron Microscopy Sciences). After the gel solidified, SVEC4-10 cells (1 × 10^5^) were added in 500 μL of DMEM supplemented with 10% FBS. The cells were incubated at 37 °C in a humidified atmosphere containing 5% CO_2_ in air for 5 h to allow for tube formation.

#### 4.7.1. Scanning Electron Microscopy

For scanning electron microscopy, after tube formation, the samples were rinsed in warm PBS (37 °C) and fixed in 2% glutaraldehyde (Electron Microscopy Sciences) in warm PBS for 2 h at room temperature. Samples were post fixed in 1% OsO_4_ (Electron Microscopy Sciences) for 2 h, rinsed in Milli-Q water (Millipore Co., Burlington, MA, USA). The samples were dehydrated in a graded ethanol series and critically point-dried with liquid CO_2_ in a Tousimis Autosandri-810 critical point dryer (Tousimis Research Co., Rockville, MD, USA), mounted on aluminum stubs with silver paint (Electron Microscopy Sciences), and coated with gold in a BAL-TEC SCD 050 Sputter Coater (BAL-TEC AG, Balzers, Liechtenstein). Samples were examined with a JEOL JSM-6610LV scanning electron microscope (JEOL, Ltd., Tokyo, Japan).

#### 4.7.2. Transmission Electron Microscopy

For transmission electron microscopy, after tube formation, the samples were rinsed in warm PBS (37 °C) and fixed in 2% glutaraldehyde (Electron Microscopy Sciences) 2% paraformaldehyde (Electron Microscopy Sciences) in 0.1 M cacodylate buffer, pH 7.4. The samples were post fixed in 1% OsO_4_ (Electron Microscopy Sciences) in 0.1 M cacodylate buffer (pH 7.4) for 2 h, rinsed in Milli-Q water, dehydrated in a graded ethanol series. The Geltrex^®^ with cells was removed from the coverslips and embedded in EMbed 812 Resin (Electron Microscopy Sciences). To demonstrate the extracellular space and mark gap junctions, 1% lanthanum nitrate was added to the fixatives. Thin sections were cut with a diamond knife and mounted on copper grids. The grids were stained for 10 min each in Reynolds’s lead citrate and 0.5% aqueous uranyl acetate. The samples were examined with a JEOL JEM-100CXII (JEOL Ltd., Tokyo, Japan) transmission electron microscope.

### 4.8. Immunoblotting

Supernatants (20 μg) from the tube formation assay from the co-cultures of SVEC4-10 cells with P815 mast cells or P815 cell lysates were boiled for 5 min in SDS sample buffer (50 mM Tris-HCl pH 6.8, 12.5% glycerol, 1% sodium dodecylsulfate, 0.01% bromophenol blue), and applied to 10% polyacrylamide gels. Proteins were then separated electrophoretically and transferred to Hybond membranes (GE Healthcare, Bio-Sciences Corp., Piscataway, NJ, USA). Membranes were blocked in TBS (0.05 M Tris–HCl, 0.15 M NaCl, pH 7.5, and 0.05% Tween 20) containing 5% nonfat dry milk. Membranes were then incubated with anti-protease antibodies (rabbit anti-mouse mMCP-5 and rabbit anti-mouse mMCP-6) were kindly provided by Michael F. Gurish (Brigham and Women’s Hospital, Harvard Medical School, Cambridge, MA, USA) at 1:200, for 1 h at room temperature, washed in TBS/Tween and incubated with goat anti-rabbit IgG conjugated to HRP (1:20,000) (Jackson ImmunoResearch, Fort Washington, PA, USA) for 30 min at room temperature, washed and developed using chemiluminescence (ECL-GE Healthcare). The blots were scanned, and the optical densities of the bands were calculated using Adobe Photoshop (Adobe Systems, San Jose, CA, USA). For the experiment control, we used a lysate from P815 cells. The cells were lysed using lysis buffer (10 mM HEPES (4-(2-hydroxyethyl)-1-piperazineethanesulfonic acid.

pH7.9, 1.5 mM MgCl_2_, 10 mM KCl, 1 mM EDTA, 10% glycerol, 1% NP-40, and 15 μL of protease inhibitor cocktail (Sigma-Aldrich, St. Louis, MO, USA).

### 4.9. β-Hexosamidase Assay

Mast cell degranulation was assessed by measuring the activity of β-hexosaminidase released into the medium of the co-cultures of SVEC4-10 cells with P815 mast cells at the end of the tube formation assay. After 5 h of co-culture, the supernatants were collected and analyzed. For the experiment control, lysates from P815 cells were used. β-hexosaminidase activity was quantified in the supernatants of co-culture and cell lysates by spectrophotometric analysis of hydrolysis of 4-Nitrophenyl *N*-acetyl-β-d-glucosaminide (Sigma-Aldrich) as previously described [20].

### 4.10. Statistical Analysis

The values are expressed as the mean ± SD. The data is expressed as mean ± SD from at least three independent experiments. In order to compare data, Student’s *t*-test was used. *p*-Values of ≤0.05 were considered significant.

## 5. Conclusions

Taken together, the results of the current study demonstrate that mast cells have both direct and indirect proangiogenic effects on endothelial cells. This investigation provides new insights into the role of mast cells in angiogenesis.

## Figures and Tables

**Table 1:** Angiogenic factors released in the presence or absence of P815 mast cells.

Angiogenic Factor	SVEC4-10 Cells	SVEC4-10 + P815 Cells	Angiogenic Factor	SVEC4-10 Cells	SVEC4-10 + P815 Cells
ADAMTS1	−	+	MMP-8	−	+
Amfiregulin	−	+	MMP-9	−	+
Angiogenin	+	+	IGFBP-9	−	+
Coagulation III	−	+	Osteopontin	−	+
CXCL16	−	+	PDGF-AA	−	+
DPPIV/CD26	−	+	PDGF	−	+
Endostatin	+	+	Pentraxin	−	+
Endothelin 1	−	+	PF 4	+	+
FGF-1	−	+	PlGF-2	−	+
FGF-2	−	+	Prolactin	−	+
Hepatopoietin A	−	+	Proliferin	−	+
IGFBP-1	−	+	SDF-1 CXCL12	−	+
IGFBP-2	−	+	Serpin E1	−	+
IGFBP-3	+	+	Serpin F1	−	+
IL-1	−	+	Trombospondin-2	−	+
Leptin	−	+	TIMP-1	−	+
MCP-1	+	+	TIMP-4	−	+
MIP-1	−	+	VEGF VPF	−	+
MMP-3	−	+	VEGF-B VRF	−	+

Abbreviations: ADAMTS1 (A disintegrin and metalloproteinase with thrombospondin motif); CD26 (Cluster of differentiation 26); DPPIV (Dipeptidyl peptidase IV); FGF (Fibroblast growth factor); IGFBP (Insulin-like Growth Factor Binding Protein); MCP (Monocyte chemoattractant protein); MIP (Macrophage inflammatory protein); MMP (matrix metalloproteinase); PDGF (Platelet derived growth factor); PF (Platelet factor); PIGF (Placental growth factor); SDF (Stromal cell-derived factor); TIMP (Tissue Inhibitor of Metalloproteinase); VEGF (Vascular endothelial growth factor); VPF (Vascular permeability factor).

## Supplementary Materials

Supplementary materials can be found at www.mdpi.com/1422-0067/18/12/2674/s1.

## References

[1] Baer C., Squadrito M.L., Iruela-Arispe M.L., de Palma M. (2013). Reciprocal interactions between endothelial cells and macrophages in angiogenic vascular niches. Exp. Cell Res..

[2] Khodarev N.N., Yu J., Labay E., Darga T., Brown C.K., Mauceri H.J., Yassari R., Gupta N., Weichselbaum R.R. (2003). Tumour-endothelium interactions in co-culture: Coordinated changes of gene expression profiles and phenotypic properties of endothelial cells. J. Cell Sci..

[3] Zhang X., Groopman J.E., Wang J.F. (2005). Extracellular matrix regulates endothelial functions through interaction of VEGFR-3 and integrin α5β1. J. Cell Physiol..

[4] Kreuger J., Phillipson M. (2016). Targeting vascular and leukocyte communication in angiogenesis, inflammation and fibrosis. Nat. Rev. Drug Discov..

[5] Freudenberg U., Zieris A., Chwalek K., Tsurkan M.V., Maitz M.F., Atallah P., Levental K.R., Eming S.A., Werner C. (2015). Heparin desulfation modulates VEGF release and angiogenesis in diabetic wounds. J. Control Release.

[6] Liebner S., Cavallaro U., Dejana E. (2006). The multiple languages of endothelial cell-to-cell communication. Arterioscler. Thromb. Vasc. Biol..

[7] Dalamon V., Fiori M.C., Figueroa V.A., Oliva C.A., del Rio R., Gonzalez W., Canan J., Elgoyhen A.B., Altenberg G.A., Retamal M.A. (2016). Gap-junctional channel and hemichannel activity of two recently identified connexin 26 mutants associated with deafness. Pflugers Arch..

[8] Figueroa X.F., Duling B.R. (2009). Gap junctions in the control of vascular function. Antioxid. Redox Signal..

[9] Bazzoni G., Dejana E. (2004). Endothelial cell-to-cell junctions: Molecular organization and role in vascular homeostasis. Physiol. Rev..

[10] Leithe E. (2016). Regulation of connexins by the ubiquitin system: Implications for intercellular communication and cancer. Biochim. Biophys. Acta.

[11] Oviedo-Orta E., Howard Evans W. (2004). Gap junctions and connexin-mediated communication in the immune system. Biochim. Biophys. Acta.

[12] Radeva M.Y., Waschke J. (2017). Mind the gap: Mechanisms regulating the endothelial barrier. Acta Physiol..

[13] Thuringer D., Boucher J., Jego G., Pernet N., Cronier L., Hammann A., Solary E., Garrido C. (2016). Transfer of functional micrornas between glioblastoma and microvascular endothelial cells through gap junctions. Oncotarget.

[14] Wang D.G., Zhang F.X., Chen M.L., Zhu H.J., Yang B., Cao K.J. (2014). Cx43 in mesenchymal stem cells promotes angiogenesis of the infarcted heart independent of gap junctions. Mol. Med. Rep..

[15] Wong P., Laxton V., Srivastava S., Chan Y.W., Tse G. (2017). The role of gap junctions in inflammatory and neoplastic disorders. Int. J. Mol. Med..

[16] Glass A.M., Snyder E.G., Taffet S.M. (2015). Connexins and pannexins in the immune system and lymphatic organs. Cell Mol. Life Sci..

[17] Vliagoftis H., Ebeling C., Ilarraza R., Mahmudi-Azer S., Abel M., Adamko D., Befus A.D., Moqbel R. (2014). Connexin 43 expression on peripheral blood eosinophils: Role of gap junctions in transendothelial migration. Biomed. Res. Int..

[18] Danese S., Sans M., de la Motte C., Graziani C., West G., Phillips M.H., Pola R., Rutella S., Willis J., Gasbarrini A. (2006). Angiogenesis as a novel component of inflammatory bowel disease pathogenesis. Gastroenterology.

[19] Bremnes R.M., Dønnem T., Al-Saad S., Al-Shibli K., Andersen S., Sirera R., Camps C., Marinez I., Busund L.T. (2011). The role of tumor stroma in cancer progression and prognosis: Emphasis on carcinoma-associated fibroblasts and non-small cell lung cancer. J. Thorac. Oncol..

[20] Jamur M.C., Grodzki A.C., Berenstein E.H., Hamawy M.M., Siraganian R.P., Oliver C. (2005). Identification and characterization of undifferentiated mast cells in mouse bone marrow. Blood.

[21] Soucie E., Brenet F., Dubreuil P. (2015). Molecular basis of mast cell disease. Mol. Immunol..

[22] Da Silva E.Z., Jamur M.C., Oliver C. (2014). Mast cell function: A new vision of an old cell. J. Histochem. Cytochem..

[23] Krystel-Whittemore M., Dileepan K.N., Wood J.G. (2015). Mast cell: A multi-functional master cell. Front. Immunol..

[24] Gilfillan A.M., Austin S.J., Metcalfe D.D. (2011). Mast cell biology: Introduction and overview. Adv. Exp. Med. Biol..

[25] Jamur M.C., Oliver C. (2011). Origin, maturation and recruitment of mast cell precursors. Front. Biosci..

[26] Zhi X., Xu C., Zhang H., Tian D., Li X., Ning Y., Yin L. (2013). Tryptase promotes atherosclerotic plaque haemorrhage in ApoE-/-mice. PLoS ONE.

[27] Blair R.J., Meng H., Marchese M.J., Ren S., Schwartz L.B., Tonnesen M.G., Gruber B.L. (1997). Human mast cells stimulate vascular tube formation. Tryptase is a novel, potent angiogenic factor. J. Clin. Investig..

[28] Wang H.W., Tedla N., Lloyd A.R., Wakefield D., McNeil P.H. (1998). Mast cell activation and migration to lymph nodes during induction of an immune response in mice. J. Clin. Investig..

[29] De Souza D.A., Borges A.C., Santana A.C., Oliver C., Jamur M.C. (2015). Mast cell proteases 6 and 7 stimulate angiogenesis by inducing endothelial cells to release angiogenic factors. PLoS ONE.

[30] Białas M., Dyduch G., Szpor J., Demczuk S., Okoń K. (2012). Microvascular density and mast cells in benign and malignant pheochromocytomas. Pol. J. Pathol..

[31] Dyduch G., Kaczmarczyk K., Okoń K. (2012). Mast cells and cancer: Enemies or allies?. Pol. J. Pathol..

[32] Cimpean A.M., Tamma R., Ruggieri S., Nico B., Toma A., Ribatti D. (2017). Mast cells in breast cancer angiogenesis. Crit. Rev. Oncol. Hematol..

[33] Varricchi G., Galdiero M.R., Loffredo S., Marone G., Iannone R., Granata F. (2017). Are mast cells masters in cancer?. Front. Immunol..

[34] Ribatti D., Ranieri G. (2015). Tryptase, a novel angiogenic factor stored in mast cell granules. Exp. Cell Res..

[35] Mondal S.K., Dasgupta S., Mandal P.K., Chatterjee S., Chakraborty D. (2014). Is there any role of mast cell density and microvessel density in cervical squamous cell carcinoma? A histologic study with special reference to cd-34 immunomarker staining. Indian J. Med. Paediatr. Oncol..

[36] Dalton D.K., Noelle R.J. (2012). The roles of mast cells in anticancer immunity. Cancer Immunol. Immunother..

[37] Jiang L., Hua Y., Shen Q., Ding S., Jiang W., Zhang W., Zhu X. (2013). Role of mast cells in gynecological neoplasms. Front. Biosci..

[38] Crivellato E., Nico B., Ribatti D. (2008). Mast cells and tumour angiogenesis: New insight from experimental carcinogenesis. Cancer Lett..

[39] Johnson C., Huynh V., Hargrove L., Kennedy L., Graf-Eaton A., Owens J., Trzeciakowski J.P., Hodges K., DeMorrow S., Han Y. (2016). Inhibition of mast cell-derived histamine decreases human cholangiocarcinoma growth and differentiation via c-Kit/stem cell factor-dependent signaling. Am. J. Pathol..

[40] Marinaccio C., Ingravallo G., Gaudio F., Perrone T., Nico B., Maoirano E., Specchia G., Ribatti D. (2014). Microvascular density, CD68 and tryptase expression in human diffuse large B-cell lymphoma. Leuk. Res..

[41] DeCicco-Skinner K.L., Henry G.H., Cataisson C., Tabib T., Gwilliam J.C., Watson N.J., Bullwinkle E.M., Falkenburg L., O’Neill R.C., Morin A. (2014). Endothelial cell tube formation assay for the in vitro study of angiogenesis. J. Vis. Exp..

[42] Arnaoutova I., Kleinman H.K. (2010). In vitro angiogenesis: Endothelial cell tube formation on gelled basement membrane extract. Nat. Protoc..

[43] Sunderkötter C., Steinbrink K., Goebeler M., Bhardwaj R., Sorg C. (1994). Macrophages and angiogenesis. J. Leukoc. Biol..

[44] Nucera S., Biziato D., de Palma M. (2011). The interplay between macrophages and angiogenesis in development, tissue injury and regeneration. Int. J. Dev. Biol..

[45] Coffelt S.B., Hughes R., Lewis C.E. (2009). Tumor-associated macrophages: Effectors of angiogenesis and tumor progression. Biochim. Biophys. Acta.

[46] Cheng L.E., Hartmann K., Roers A., Krummel M.F., Locksley R.M. (2013). Perivascular mast cells dynamically probe cutaneous blood vessels to capture immunoglobulin E. Immunity.

[47] De Almeida Buranello P.A., Moulin M.R., Souza D.A., Jamur M.C., Roque-Barreira M.C., Oliver C. (2010). The lectin artinm induces recruitment of rat mast cells from the bone marrow to the peritoneal cavity. PLoS ONE.

[48] Bauer A.L., Jackson T.L., Jiang Y. (2007). A cell-based model exhibiting branching and anastomosis during tumor-induced angiogenesis. Biophys. J..

[49] Song J.W., Bazou D., Munn L.L. (2012). Anastomosis of endothelial sprouts forms new vessels in a tissue analogue of angiogenesis. Integr. Biol..

[50] Diaz-Santana A., Shan M., Stroock A.D. (2015). Endothelial cell dynamics during anastomosis in vitro. Integr. Biol..

[51] Fantin A., Vieira J.M., Gestri G., Denti L., Schwarz Q., Prykhozhij S., Peri F., Wilson S.W., Ruhrberg C. (2010). Tissue macrophages act as cellular chaperones for vascular anastomosis downstream of VEGF-mediated endothelial tip cell induction. Blood.

[52] Figueroa X.F., Isakson B.E., Duling B.R. (2004). Connexins: Gaps in our knowledge of vascular function. Physiology.

[53] Brink P.R., Ricotta J., Christ G.J. (2000). Biophysical characteristics of gap junctions in vascular wall cells: Implications for vascular biology and disease. Braz. J. Med. Biol. Res..

[54] Leithe E., Mesnil M., Aasen T. (2017). The connexin 43 C-terminus: A tail of many tales. Biochim. Biophys. Acta.

[55] Gleisner M.A., Navarrete M., Hofmann F., Salazar-Onfray F., Tittarelli A. (2017). Mind the gaps in tumor immunity: Impact of connexin-mediated intercellular connections. Front. Immunol..

[56] Kwak B.R., Mulhaupt F., Veillard N., Gros D.B., Mach F. (2002). Altered pattern of vascular connexin expression in atherosclerotic plaques. Arterioscler. Thromb. Vasc. Biol..

[57] Polacek D., Bech F., McKinsey J.F., Davies P.F. (1997). Connexin43 gene expression in the rabbit arterial wall: Effects of hypercholesterolemia, balloon injury and their combination. J. Vasc. Res..

[58] Vliagoftis H., Hutson A.M., Mahmudi-Azer S., Kim H., Rumsaeng V., Oh C.K., Moqbel R., Metcalfe D.D. (1999). Mast cells express connexins on their cytoplasmic membrane. J. Allergy Clin. Immunol..

[59] Figueroa X.F., Isakson B.E., Duling B.R. (2006). Vascular gap junctions in hypertension. Hypertension.

[60] Pistorio A.L., Ehrlich H.P. (2011). Modulatory effects of connexin-43 expression on gap junction intercellular communications with mast cells and fibroblasts. J. Cell Biochem..

[61] Au S.R., Au K., Saggers G.C., Karne N., Ehrlich H.P. (2007). Rat mast cells communicate with fibroblasts via gap junction intercellular communications. J. Cell Biochem..

[62] Foley T.T., Saggers G.C., Moyer K.E., Ehrlich H.P. (2011). Rat mast cells enhance fibroblast proliferation and fibroblast-populated collagen lattice contraction through gap junctional intercellular communications. Plast. Reconstr. Surg..

[63] De Souza Junior D.A., Santana A.C., da Silva E.Z., Oliver C., Jamur M.C. (2015). The role of mast cell specific chymases and tryptases in tumor angiogenesis. Biomed. Res. Int..

[64] Ribatti D., Ranieri G., Nico B., Benagiano V., Crivellato E. (2011). Tryptase and chymase are angiogenic in vivo in the chorioallantoic membrane assay. Int. J. Dev. Biol..

[65] Distler J.H., Hirth A., Kurowska-Stolarska M., Gay R.E., Gay S., Distler O. (2003). Angiogenic and angiostatic factors in the molecular control of angiogenesis. Q. J. Nucl. Med..

[66] Dunn T.B., Potter M. (1957). A transplantable mast-cell neoplasm in the mouse. J. Natl. Cancer Inst..

[67] Furuno T., Teshima R., Kitani S., Sawada J., Nakanishi M. (1996). Surface expression of CD63 antigen (AD1 antigen) in P815 mastocytoma cells by transfected IgE receptors. Biochem. Biophys. Res. Commun..

[68] Miller L., Alber G., Varin-Blank N., Ludowyke R., Metzger H. (1990). Transmembrane signaling in P815 mastocytoma cells by transfected ige receptors. J. Biol. Chem..

[69] Schindler R., Day M., Fischer G.A. (1959). Culture of neoplastic mast cells and their synthesis of 5-hydroxytryptamine and histamine in vitro. Cancer Res..

[70] Khoo C.P., Micklem K., Watt S.M. (2011). A comparison of methods for quantifying angiogenesis in the matrigel assay in vitro. Tissue Eng. Part C Methods.

[71] Lee W.S., Park Y.L., Kim N., Oh H.H., Son D.J., Kim M.Y., Oak C.Y., Chung C.Y., Park H.C., Kim J.S. (2015). Myeloid cell leukemia-1 is associated with tumor progression by inhibiting apoptosis and enhancing angiogenesis in colorectal cancer. Am. J. Cancer Res..

[72] Askou A.L., Aagaard L., Kostic C., Arsenijevic Y., Hollensen A.K., Bek T., Jensen T.G., Mikkelsen J.G., Corydon T.J. (2015). Multigenic lentiviral vectors for combined and tissue-specific expression of miRNA- and protein-based antiangiogenic factors. Mol. Ther. Methods Clin. Dev..

