# Supplementary material for: Mast Cells Interact with Endothelial Cells to Accelerate In Vitro Angiogenesis
Source: Int J Mol Sci. 2017 Dec 13;18(12):2674. doi: 10.3390/ijms18122674 (PMC5751276; doi:10.3390/ijms18122674)
Supplement: Supplementary file 1 [file ijms-18-02674-s001.pdf]

A

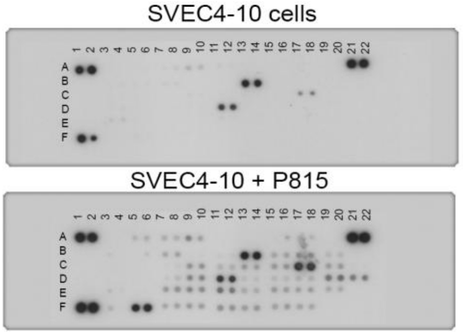

B

Coordinate	Analyte/Control	Coordinate	Analyte/Control
A1, A2	Reference Spots	D5, D6	IP-10
A5, A6	ADAMTS1	D7, D8	KC
A7, A8	Amphiregulin	D9, D10	Leptin
A9, A10	Angiogenin	D11, D12	MCP-1
A11, A12	Angiopoietin-1	D13, D14	MIP-1α
A13, A14	Angiopoietin-3	D15, D16	MMP-3
A15, A16	Coag. Factor III	D17, D18	MMP-8
A17, A18	CXCL16	D19, D20	MMP-9
A21, A22	Reference Spots	D21, D22	NOV
B3, B4	Cyr61	E3, E4	Osteopontin
B5, B6	DLL4	E5, E6	PD-ECGF
B7, B8	DPPIV	E7, E8	PDGF-AA
B9, B10	EGF	E9, E10	PDGF-AB
B11, B12	Endoglin	E11, E12	Pentraxin-3
B13, B14	Endostatin	E13, E14	Platelet Factor 4
B15, B16	Endothelin-1	E15, E16	PlGF-2
B17, B18	FGF acidic	E17, E18	Prolactin
B19, B20	FGF basic	E19, E20	Proliferin
C3, C4	KGF	F1, F2	Reference Spots
C5, C6	Fractalkine	F3, F4	SDF-1
C7, C8	GM-CSF	F5, F6	Serpin E1
C9, C10	HB-EGF	F7, F8	Serpin F1
C11, C12	HGF	F9, F10	Thrombospondin-2
C13, C14	IGFBP-1	F11, F12	TIMP-1
C15, C16	IGFBP-2	F13, F14	TIMP-4
C17, C18	IGFBP-3	F15, F16	VEGF
C19, C20	IL-1α	F17, F18	VEGF-B
C21, C22	IL-1β	F19, F20	Negative Control
D3, D4	IL-10		

Figure S1. Differential release of angiogenic factors by SVEC4-10 cells in the presence or absence of mast cells. The SVEC4-10 cells were cultured for 5 hours at 37 °C on Geltrex® in the presence or absence of P815 mast cells. Culture supernatants were used to analyze the protein expression of pro- and anti-angiogenic factors using the Proteome Profiler™ Mouse Angiogenesis Array Kit. **(A**) Array membrane images are shown. (**B**) Table giving the mouse angiogenesis array coordinates with a description and location of each angiogenic factor in the membrane array.

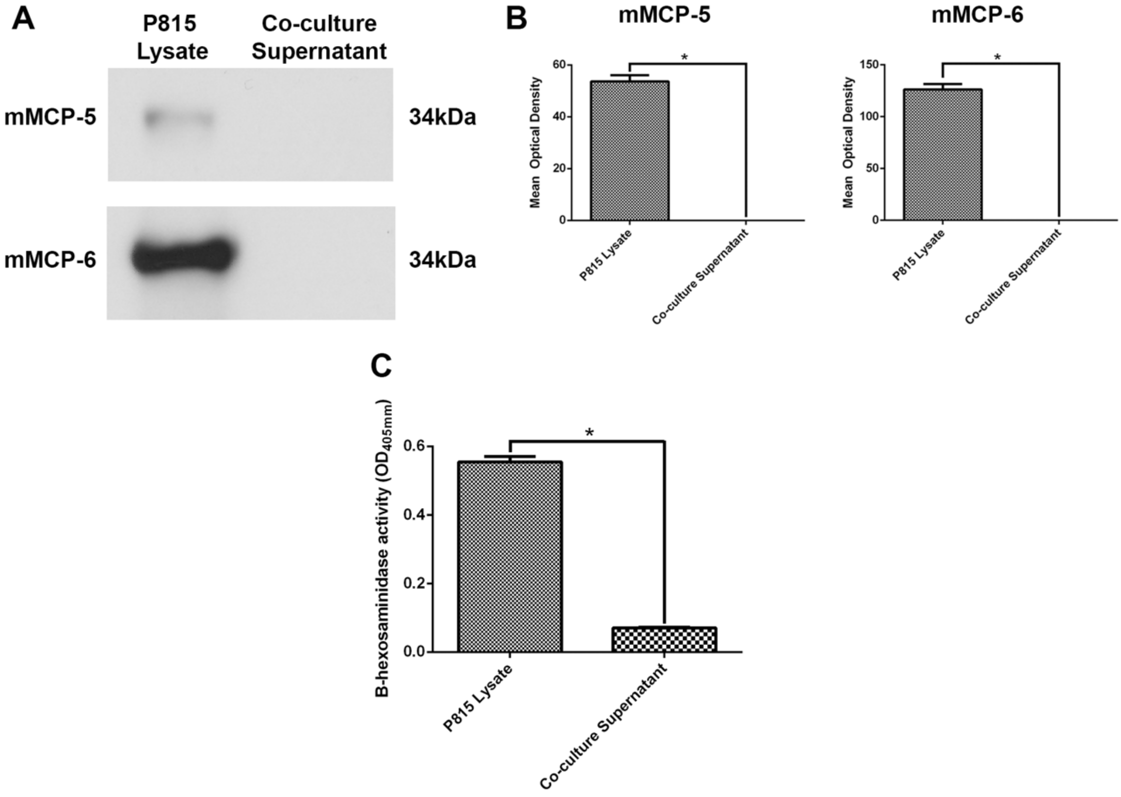

Figure S2. No chymase (mMCP-5) or tryptase (mMCP-6) expression or β-hexosaminidase activity was detected in the supernatants from the co-culture. (A) Western blots of cell lysates or supernatants from the tube formation assay from the co-cultures of SVEC4-10 cells with P815 mast cells or the lysate of P815 cells. The expression of mMPC-5 and mMCP-6 is not detectable in the supernatants from the co-cultures during tube formation assay. (B) The graphs shown the mean of optical density of the protein bands from 3 independent experiments. (C) No β-hexosaminidase activity was detected in the supernatants from the co-culture. The β-hexosaminidase activity in the co-culture supernatant is similar to that of the blank control. *$p \leq 0.05$.
